# Automated smoother for the numerical decoupling of dynamics models

**DOI:** 10.1186/1471-2105-8-305

**Published:** 2007-08-21

**Authors:** Marco Vilela, Carlos CH Borges, Susana Vinga, Ana Tereza R Vasconcelos, Helena Santos, Eberhard O Voit, Jonas S Almeida

**Affiliations:** 1 Dept. Computatinal and Applied Mathematics, Laboratório Nacional de Computação Científica, Petrópolis, Rio de Janeiro, Brazil; 2 Instituto de Tecnologia Química e Biológica, Universidade Nova de Lisboa, Rua da Quinta Grande 6, Apartado 127, 2780-156 Oeiras, Portugal; 3 Instituto de Engenharia de Sistemas e Computadores: Investigação e Desenvolvimento (INESC-ID), R. Alves Redol 9, 1000-029 Lisboa, Portugal; 4 Dept. Biomedical Engineering, Georgia Institute of Technology, Atlanta, GA 30332, USA; 5 Dept. Bioinformatics and Computational Biology, M. D. Anderson Cancer Center, 1515 Holcombe Blvd, Houston, TX 77030, USA

## Abstract

**Background:**

Structure identification of dynamic models for complex biological systems is the cornerstone of their reverse engineering. Biochemical Systems Theory (BST) offers a particularly convenient solution because its parameters are kinetic-order coefficients which directly identify the topology of the underlying network of processes. We have previously proposed a numerical decoupling procedure that allows the identification of multivariate dynamic models of complex biological processes. While described here within the context of BST, this procedure has a general applicability to signal extraction. Our original implementation relied on artificial neural networks (ANN), which caused slight, undesirable bias during the smoothing of the time courses. As an alternative, we propose here an adaptation of the Whittaker's smoother and demonstrate its role within a robust, fully automated structure identification procedure.

**Results:**

In this report we propose a robust, fully automated solution for signal extraction from time series, which is the prerequisite for the efficient reverse engineering of biological systems models. The Whittaker's smoother is reformulated within the context of information theory and extended by the development of adaptive signal segmentation to account for heterogeneous noise structures. The resulting procedure can be used on arbitrary time series with a nonstationary noise process; it is illustrated here with metabolic profiles obtained from *in-vivo *NMR experiments. The smoothed solution that is free of parametric bias permits differentiation, which is crucial for the numerical decoupling of systems of differential equations.

**Conclusion:**

The method is applicable in signal extraction from time series with nonstationary noise structure and can be applied in the numerical decoupling of system of differential equations into algebraic equations, and thus constitutes a rather general tool for the reverse engineering of mechanistic model descriptions from multivariate experimental time series.

## Background

The reverse engineering of biological systems from experimental data often cannot be achieved on first principles. This is as much a reflection of the lack of plausible hypotheses as it is an indication of excessive parametric sensitivity when alleged mechanistic formulations are at hand. Consequently, there is a critical need for a description of dynamic behaviors that can be used as a machine learning tool (*e.g.*, as a generic "black box"), but with parameters capable of shedding light on the topology of the underlying mechanisms. Biochemical Systems Theory [1-3], offers such formalism, especially in the form of S-systems, where kinetic-order coefficients characterize the topology of a biological network as well as the magnitude of each interaction. A drawback of this approach is that the parameterization of S-systems is a difficult problem, even for five metabolic species [4]. In a previous report [5], we proposed to overcome this challenge by tracing each species independently with a universal function of time, *x(t) = f(t)*, such that *dx/dt = g(t)*, and where *g(t) = df(t)/dt *is deduced symbolically from the neural network transfer function; see also [6]. The independency of each metabolic profile allows solving the S-system linearization problem by decoupling it, which reduces the computational effort in the system parameters identification by preventing numerical integration. In the earlier report we suggested using artificial neural networks (ANN) with optimized topology and early stopping procedures [7]. The distinctive advantage of using an ANN is that it is a universal function [8] with a closed form for which we were able to determine the first derivative symbolically [5]. The ANN solution, however, is not without problems. The most significant issue is that its discriminant function often leads to artifacts in its derivatives, which distort the solution, even when they are not visually apparent in the smoothed signal. The artifacts reflect the sigmoidal transfer function of the ANN model. That conclusion drove the pursuit of an entirely implicit solution that is driven solely by the experimental data and is free of all parametric bias.

The task of inferring signal from noisy time series falls into the general category of developing denoising filters. In an effort extracting signal from noise in chromatograms, Paul Eilers [9] recently proposed a "perfect smoother", which is basically a matrix form of a much older implicit method known as Whittaker's smoother [10]. Those works [9,10] are the starting point for the procedure introduced here. Consideration of the denoising problem as the task of "learning" an arbitrary signal suggests the possibility of applying principles from Information Theoretic Learning [11-14], which allows signal scaling without causing bias in signal extraction. Specifically, the use of quadratic Renyi's entropy for assessing the learning process offers a foundation for the re-identification of smoothers based on Error Entropy Minimization (EEM). This procedure has been successfully applied to chaotic time series prediction and in nonlinear system identification, where the mean square error was replaced by error entropy as cost function, for instance, in the training of ANN models [11]. In this report, we explore the use of error entropy as optimization criterion for reconfiguring the Whittaker's smoother. In complementary research, we, as well as many others, have been working on parameterization procedures for S-systems [*e.g.*, [15] and references therein], implicitly assuming that noisy time series and their slopes could efficiently be smoothed by some unidentified procedure. The algorithm reported here describes such a procedure.

## Results

### Metabolic profiles

The proposed signal extraction method was tested with an application to metabolic profiles. These had been measured with *in vivo *NMR methods at a sequence of time points and quantify glycolysis in the lactic acid producing bacterium *Lactococcus lactis *[16]. The experimental data are very interesting because the underlying molecular mechanisms are relatively well understood, because of the high frequency of sampling, and because the data have a complicated time course and noise structure. They were therefore recently proposed as a reference case study for testing reverse engineering methods for biological networks [17]. The data were included in our open source MATLAB toolbox and stand-alone GUI application, described in the Section *Availability and requirements*.

**Figure 3:**
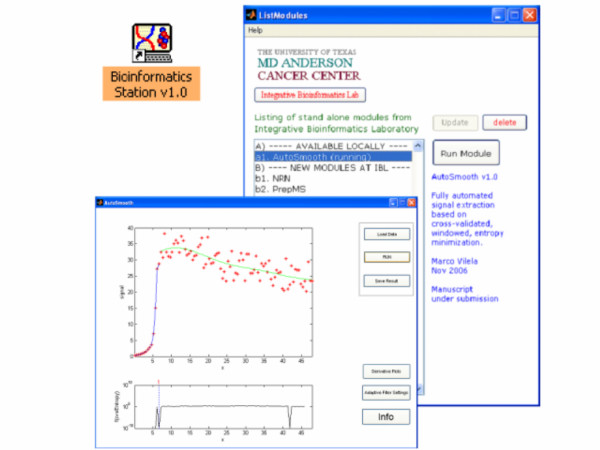
**Software application**. Snapshot of accompanying AutoSmooth application. The application and algorithm are provided both as open source (Matlab) code and as stand-alone applications that can be used without requiring commercial licenses. The application can also be managed conveniently as a *BioinformaticStation.org *module.

In addition to the data analysis described above, the performance of the proposed filter was evaluated for simulated data, in an effort to assess its ability to detect and filter different noise structures correctly. These tests included the processing of noise-free signals, where the smoothing procedure succeeded in automatically determining splines with appropriate order. These and other tests can be verified by either running the Matlab code, provided as a open-source, in the appropriate programming environment or using the corresponding stand-alone (compiled executable), public domain application, for which no commercial license is needed (Figure 3).

## Discussion

In this report, Rényi's second-order entropy of the cross-validation error entropy (*cvEE*) was used as optimization criterion for the parameters estimation in Whittaker's smoother. The optimization process is based on gradient ascent of the information potential of *cvE *in *λ *direction, where Parzen Windows with Gaussian kernel are used for the *pdf *estimation. In general, this type of *pdf *estimation faces one particular problem: the kernel size *σ*. This variable is crucial for convergence of the gradient method because it causes the algorithm to reach an optimal local minimal if its value is misestimated. The estimation of kernel size from the data covariance has yielded good results in some applications [18]. In our software, the user can choose an alternative automatic method that uses a machine learning kernel [19] or set the value of *σ *manually. The effect of this parameter for specific data sets can therefore be studied by systematically screening a range of values for the particular application. Although we found the automatic setting to be generally satisfactory, this is only an empirical result, which suggests that *pdf *estimation warrants further investigation.

**Figure 1:**
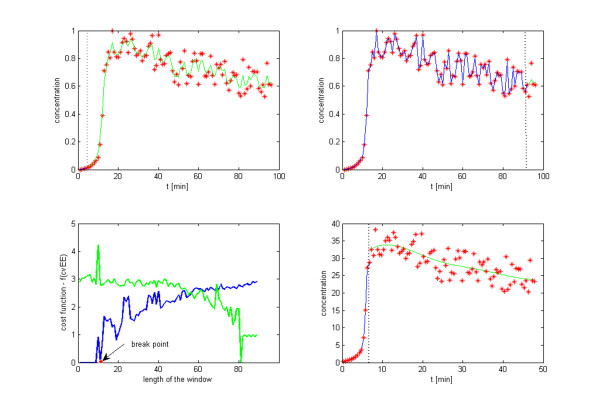
**Scanning process**. Beginning of the first scanning, where the breakpoint (dotted line) segments the first four time points from the rest of the signal. b) End of the first scan, when the breakpoint separates the last four time points from the rest of the signal. c) Cost function of all scanned window partitions. The optimal break point is marked with an arrow. d) Signal extraction by the optimal window partition. The scanning process is now repeated for each of the two windows individually. The two windows represent optimal partitions of signals with distinct noise structures. Therefore, the optimal values of *d *and *λ *identified for each window reflect that distinction, are respectively 4 and 1 for the pre-partition signal and 4 and 10^6.7525 ^for the post-partition signal.

**Figure 2:**
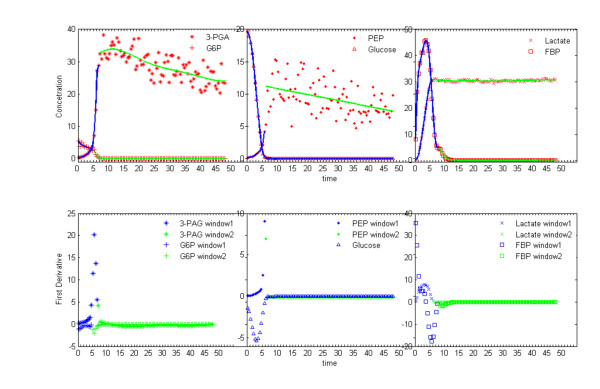
**Result in real data**. Illustration of the smoothing procedure applied to *in vivo Lactococcus lactis *time series for Glucose, Glucose 6-phosphate (G6P), Fructose 1,6-bisphosphate (FBP), 3-Phosphoglycerate (3-PGA), Phosphoenolpyruvate (PEP), and Lactate [16]. The first derivative is shown below the corresponding metabolic time series. The window partitions are shown with distinct colors. It is noteworthy that the shift in noise structure, which segments the signal into smaller temporal windows with noise invariance, is approximately the same for all metabolites except FBP. Since the smoothing procedure is applied independently to each metabolite, this coupling suggests shared dependency on some molecular machinery, which changes when its main substrate, Glucose, is depleted at t ~ 6 min.

The application of the proposed combined procedures (adaptive smoother and the segmentation algorithm) described in the Methods Section to the *Lactococcus *data demonstrates the quality of the smoothing algorithm (Figures 1 and 2). Most impressive may be that these results were obtained in a fully automated fashion. As we suspected in previous analyses of biological systems and their reverse engineering [5,16,17], the results here suggest that the structure of the noise appears to be specific to distinct phases of the molecular machinery underlying the observed behavior. For example, Figure 2 clearly indicates that depletion of glucose is associated with relatively little noise in the signal from intermediate metabolites (Figure 2 before t < 6 min). The exhaustion of substrate (t ~ 6 min), however, triggers a synchronized effect in all but one of the metabolites, which results in a marked increase in noise and is reflected by the synchronized break point in the corresponding window segmentations. Since the analytical method (*in vivo *NMR) does not change and the window segmentation procedure is applied to each metabolite independently, the sharp increase in signal noise may be best interpreted as a change in the functioning of the glycolytic machinery, which for instance could be mediated by a shift in the NADH/NAD ratio [1]. The synchronized succession of distinct periods with relatively invariant noise structure may be specific to biological processes, where a succession of dynamic behaviors is often associated with a shift between physiological states. The windowing approach reveals these shifts.

It should be noted that the reliance on cross-validation implies that time points at the edges of each window cannot be used for signal extraction. This loss at break points might appear to be a significant drawback of the filtering procedure. However, as established in prior work [6], the identification of decoupled systems is discontinuous in nature as it consist on the generation of pairs of (*dx/dt*, *x*) values. Therefore, it suffers only mildly from a few missing or omitted data points. More important is that multiple independent time series are available to narrow the boundary estimates for the system parameters to the point where the topology of the biological network can be inferred with reliability.

## Conclusion

The goal of developing a "perfect smoother" that can be used as an automated tool for signal extraction has been an elusive goal in the field of signal processing. Based on historical work that started with the Whittaker's smoother and was advanced by cross-validation in Eilers' smoother, here we take that approach one step further by removing the parametric bias of using squared deviations as an optimization criterion. In its place we proposed an informational measure of variation in the form of cross-validated error entropy. The crucial step of the proposed methodology is the identification of the matrix format of the *cvE *(Equation 12) that permits a closed-form solution for its derivative with respect to the smoothing parameter *λ *(Equation 15). That solution is also used to segment the signal in windows where the consideration of the neighboring values would decrease the optimality of within-window signal extraction. The resulting algorithm is fully automated and was successfully applied to reference, notoriously difficult biological time series. Applicability to signal extraction in other areas may be anticipated.

## Methods

### Starting point: Whittaker's smoother

The well-known Whittaker's smoother [10] was formulated to fit a smooth series *z *to a given series *y *of *N *noisy data points. The task was proposed as minimization of the following function:

Q=∑i=1N(yi−zi)2+λ∑i=1N−d(Δzi)2
 MathType@MTEF@5@5@+=feaafiart1ev1aaatCvAUfKttLearuWrP9MDH5MBPbIqV92AaeXatLxBI9gBaebbnrfifHhDYfgasaacH8akY=wiFfYdH8Gipec8Eeeu0xXdbba9frFj0=OqFfea0dXdd9vqai=hGuQ8kuc9pgc9s8qqaq=dirpe0xb9q8qiLsFr0=vr0=vr0dc8meaabaqaciaacaGaaeqabaqabeGadaaakeaacqWGrbqucqGH9aqpdaaeWbqaamaabmaabaGaemyEaK3aaSbaaSqaaiabdMgaPbqabaGccqGHsislcqWG6bGEdaWgaaWcbaGaemyAaKgabeaaaOGaayjkaiaawMcaamaaCaaaleqabaGaeGOmaidaaaqaaiabdMgaPjabg2da9iabigdaXaqaaiabd6eaobqdcqGHris5aOGaey4kaSccciGae83UdW2aaabCaeaadaqadaqaaiabfs5aejabdQha6naaBaaaleaacqWGPbqAaeqaaaGccaGLOaGaayzkaaWaaWbaaSqabeaacqaIYaGmaaaabaGaemyAaKMaeyypa0JaeGymaedabaGaemOta4KaeyOeI0IaemizaqganiabggHiLdaaaa@51E7@

Equation 1 describes the balance of two additive components: one quantifying the smoothness Δ*z*_*i *_of the output data and the other quantifying the fidelity of the smoothed model output to the raw data (*y*_*i *_- *z*_*i*_). Thus, the parameterization of this smoother consists of finding an optimal weighing of the two components of *Q*. The solution is signal specific and involves the identification of optimal values for the order of the filter, *d*, and the weighing of its residuals, *λ*. The order *d *is an implicit parameter that determines the specific definition of Δ*z*_*i*_. For example, for a first order filter (*d = 1*), the smoothness measure Δ*z*_*i *_of the output data is defined as the difference between two consecutive points Δ*z*_*i *_= (*z*_*i*+1 _- *z*_*i*_). For a 2^nd ^order filter, *d = 2*, the smoothness measure is formulated as the difference between pair-wise differences among three consecutive points Δ*z*_*i *_= (*z*_*i*+2 _- *z*_*i*+1_) - (*z*_*i*+1 _- *z*_*i*_). The accumulation of differences proceeds for higher orders accordingly. The order *d *is therefore an integer number that sets the flexibility of the signal extracted. By contrast, the positive real scalar *λ *weighs the smoothness term Δ*z*_*i *_such that high values dampen the extracted signal. Accordingly, high values of *d *and low values of *λ *tend to cause over-fitting, while the inverse combination leads to more ragged output. In cases of very high values of *λ*, the filter behaves like polynomial regression of degree *d *- 1 [9]. For implementation purposes, the original proposition of a "perfect smoother" rewrites the Equation 1 in its matrix form [9], where *D *is a (*N *- *d*) × *N *matrix such that *Dz *= ∑Δ*z *and |*A*|^2 ^= ∑*A*^2^:

*Q *= |*y *- *z*|^2 ^+ *λ*|*Dz*|^2^

Minimization of Equation 2 is achieved by computing its first partial derivate with respect to *z *and solving for zero:

∂Q/∂z=−2(y−z)+2λDtDz=0z=(I+λDTD)−1y
 MathType@MTEF@5@5@+=feaafiart1ev1aaatCvAUfKttLearuWrP9MDH5MBPbIqV92AaeXatLxBI9gBaebbnrfifHhDYfgasaacH8akY=wiFfYdH8Gipec8Eeeu0xXdbba9frFj0=OqFfea0dXdd9vqai=hGuQ8kuc9pgc9s8qqaq=dirpe0xb9q8qiLsFr0=vr0=vr0dc8meaabaqaciaacaGaaeqabaqabeGadaaakeaafaqaaeGabaaabaWaaSGbaeaacqGHciITcqWGrbquaeaacqGHciITcqWG6bGEaaGaeyypa0JaeyOeI0IaeGOmaiJaeiikaGIaemyEaKNaeyOeI0IaemOEaONaeiykaKIaey4kaSIaeGOmaidcciGae83UdWMaemiraq0aaWbaaSqabeaacqWG0baDaaGccqWGebarcqWG6bGEcqGH9aqpcqaIWaamaeaacqWG6bGEcqGH9aqpcqGGOaakcqWGjbqscqGHRaWkcqWF7oaBcqWGebardaahaaWcbeqaaiabdsfaubaakiabdseaejabcMcaPmaaCaaaleqabaGaeyOeI0IaeGymaedaaOGaemyEaKhaaaaa@5482@

In the Equation 3, *I *represents the identity matrix of order *N*. The smoother equation can be written in a more general form, where the noisy time series *y *can have missing points. Let *w *be a vector of weights with the same dimension of *y *where for each missing point *y*_*i*_, *w*_*i *_= *0 *and *w*_*i *_= *1 *otherwise. Thus, Equation 3 can be rewritten as

*z *= (*W *+ *λD*^*T*^*D*)^-1^*Wy*

In the Equation 4, *W *is a diagonal matrix with *w *on its diagonal. Therefore, the extraction of a signal from a given data series consists of identifying the scalar *λ *and the integer order *d *(implicit in the matrix *D*) in the Equation 4. One method to estimate these two parameters was described in [9] as an exhaustive search for the pair-wise (*λ*, *d*) that minimizes the cross-validation error (*cvE*), easily obtained by Equation 4

ei=yi−zi1−Hii
 MathType@MTEF@5@5@+=feaafiart1ev1aaatCvAUfKttLearuWrP9MDH5MBPbIqV92AaeXatLxBI9gBaebbnrfifHhDYfgasaacH8akY=wiFfYdH8Gipec8Eeeu0xXdbba9frFj0=OqFfea0dXdd9vqai=hGuQ8kuc9pgc9s8qqaq=dirpe0xb9q8qiLsFr0=vr0=vr0dc8meaabaqaciaacaGaaeqabaqabeGadaaakeaacqWGLbqzdaWgaaWcbaGaemyAaKgabeaakiabg2da9maalaaabaGaemyEaK3aaSbaaSqaaiabdMgaPbqabaGccqGHsislcqWG6bGEdaWgaaWcbaGaemyAaKgabeaaaOqaaiabigdaXiabgkHiTiabdIeainaaBaaaleaacqWGPbqAcqWGPbqAaeqaaaaaaaa@3D85@

where *H *is the "hat matrix", given by

*H *= (*W *+ *λD*^*T*^*D*)^-1^*W*

The use of the *cvE *in an automatic Whittaker's smoother optimization was advised as a limited procedure [9]. In an early implementation using *cvE *as cost function, we found out that the problem of automation using *cvE *is also associated with the scale and skewness of the residual variation. An adaptive supervised method specifically developed for the optimization of the smoother is described in this report by identifying a novel formulation that is not sensitive to signal scaling (it is therefore non-parametric), but only to its distribution. This is an important characteristic when a comparison of an error measure between windows with different signal structures is made. Symptomatically, we have observed that the exhaustion of key metabolites like glucose will cause dramatic shifts in noise structure. Accordingly, noise restructuring was targeted as the defining feature to find window boundaries by the segmentation algorithm, as if distinguishing what could be thought as distinct signals. Despite the advantages of the new formulation, the issue of the under-smoothing with correlated data remains. This problem might be addressable by weighting the cost function with the eigenvalues of the covariance matrix as described in [20], but is beyond the scope of this report.

### Information-theoretic learning

During the past two decades, information theory has become popular for solving signal extraction problems, where a defined metric between the probability density function (*pdf*) of the input signal and the *pdf *of the output system is optimized [13,14,21,22]. In contrast to methods that rely on some second-order statistic (systems with mean-square error as optimization criterion), the *pdf *"matching" can be validly applied to non-Gaussian signal structures if one relies on high-order statistics (HOS) that characterize the signal distribution [22]. This approach is equivalent to minimization of the Kullback-Leibler distance between the *pdf's *of the input data and output system [22,23]. From the point of view of information-theoretic learning, minimization of the Csiszar distance (of which the Kullback-Leibler distance is a special case) between thepdf's of the input and output systems can be achieved by minimization of Renyi's error entropy of the system, which corresponds to the minimization of the information contained in the error [11]. Therefore, the average information recovered from a given signal with pdf *f*_*y*_*(·) *can be quantified by Renyi's second-order entropy, as defined in Equation 7, where the argument *V(y) *is called *Information Potential *(*IP*) and defined in Equation 8.

*H*_*R*2_(*y*) = -log(*V*(*y*))

V(y)=∫−∞∞fy2(ξ)dξ
MathType@MTEF@5@5@+=feaafiart1ev1aaatCvAUfKttLearuWrP9MDH5MBPbIqV92AaeXatLxBI9gBaebbnrfifHhDYfgasaacH8akY=wiFfYdH8Gipec8Eeeu0xXdbba9frFj0=OqFfea0dXdd9vqai=hGuQ8kuc9pgc9s8qqaq=dirpe0xb9q8qiLsFr0=vr0=vr0dc8meaabaqaciaacaGaaeqabaqabeGadaaakeaacqWGwbGvcqGGOaakcqWG5bqEcqGGPaqkcqGH9aqpdaWdXbqaaiabdAgaMnaaDaaaleaacqWG5bqEaeaacqaIYaGmaaGccqGGOaakiiGacqWF+oaEcqGGPaqkaSqaaiabgkHiTiabg6HiLcqaaiabg6HiLcqdcqGHRiI8aOGaemizaqMae8NVdGhaaa@42DA@

The *pdf f*_*y*_*(·) *can be numerically approximated by a kernel density function. Here, we use Parzen windows with Gaussian kernel *k*_*σ *_with size *σ *to obtain a discrete solution to the estimation of *IP*, which leads to

V(y)=1N2∑i=1N∑j=1Nk2σ(yj−yi)
 MathType@MTEF@5@5@+=feaafiart1ev1aaatCvAUfKttLearuWrP9MDH5MBPbIqV92AaeXatLxBI9gBaebbnrfifHhDYfgasaacH8akY=wiFfYdH8Gipec8Eeeu0xXdbba9frFj0=OqFfea0dXdd9vqai=hGuQ8kuc9pgc9s8qqaq=dirpe0xb9q8qiLsFr0=vr0=vr0dc8meaabaqaciaacaGaaeqabaqabeGadaaakeaacqWGwbGvcqGGOaakcqWG5bqEcqGGPaqkcqGH9aqpdaWcaaqaaiabigdaXaqaaiabd6eaonaaCaaaleqabaGaeGOmaidaaaaakmaaqahabaWaaabCaeaacqWGRbWAdaWgaaWcbaGaeGOmaidcciGae83WdmhabeaakiabcIcaOiabdMha5naaBaaaleaacqWGQbGAaeqaaOGaeyOeI0IaemyEaK3aaSbaaSqaaiabdMgaPbqabaGccqGGPaqkaSqaaiabdQgaQjabg2da9iabigdaXaqaaiabd6eaobqdcqGHris5aaWcbaGaemyAaKMaeyypa0JaeGymaedabaGaemOta4eaniabggHiLdaaaa@4FDE@

[11]. In our application, the parameters *λ *and *d *of the filter are optimized by minimizing Renyi's second-order entropy of the cross-validation error.

### Minimal cross-validation error entropy

The identification of values for *λ *and *d *in the Whittaker's smoother is challenging to the point that the original report advised against automation altogether [9]. The computational challenge is exacerbated when automation is combined with a nested estimation of the information potential, *IP *(Equations 8 and 9). To overcome these complications, the method proposed here minimizes error entropy instead of the typical mean square error (MSE), as it has been recommended for the extraction of information in adaptive systems [12]. Specifically, we propose a new method for determining optimal values for *λ *and *d *in Whittaker's smoother, where the cross-validation error entropy (*cvEE*) is used instead of the cross-validation error (*cvE*). As the logarithm is a monotonic function, the minimization of Renyi's second-order entropy is equivalent to the maximization of *IP *[12]. However, the optimization procedure has to be tailored to the integer nature of *d*, which requires a different treatment than the gradient method employed for the identification of *λ*. For this reason, the algorithm searches for *d *within a reasonable set of integer orders (between 1 and 6), and for each value of *d *the *λ *parameter is found by the gradient of *IP *as described in Equation 10:

*λ*_*i*+1 _= *λ*_*i *_+ *η*∇_*λ*_*V*(*e*)

Here, *η *is the learning rate and ***e ***represents the *cvE *vector. The adaptation of *λ *terminates when one of the stop criteria is satisfied; that is, if either the *cvE *entropy increases, or if the algorithm reaches the minimal gradient or the maximum number of epochs. The order value *d *and the *λ *value with the minimal *cvEE *are chosen as the optimal parameters values (see next Subsection).

### Gradient of cvEE

In order to optimize the value of *λ*, we propose a cross-validation error entropy method as optimization criterion. The gradient of the information potential of *cvE *in *λ *direction is given by Equation 11:

∂V(e)∂λ=12N2σ2∑i=1N∑j=1N(ej−ei)k2σ(ei,ej)[∂ei∂λ−∂ej∂λ]
 MathType@MTEF@5@5@+=feaafiart1ev1aaatCvAUfKttLearuWrP9MDH5MBPbIqV92AaeXatLxBI9gBaebbnrfifHhDYfgasaacH8akY=wiFfYdH8Gipec8Eeeu0xXdbba9frFj0=OqFfea0dXdd9vqai=hGuQ8kuc9pgc9s8qqaq=dirpe0xb9q8qiLsFr0=vr0=vr0dc8meaabaqaciaacaGaaeqabaqabeGadaaakeaadaWcaaqaaiabgkGi2kabdAfawjabcIcaOiabdwgaLjabcMcaPaqaaiabgkGi2IGaciab=T7aSbaacqGH9aqpdaWcaaqaaiabigdaXaqaaiabikdaYiabd6eaonaaCaaaleqabaGaeGOmaidaaOGae83Wdm3aaWbaaSqabeaacqaIYaGmaaaaaOWaaabCaeaadaaeWbqaaiabcIcaOiabdwgaLnaaBaaaleaacqWGQbGAaeqaaOGaeyOeI0Iaemyzau2aaSbaaSqaaiabdMgaPbqabaGccqGGPaqkcqWGRbWAdaWgaaWcbaGaeGOmaiJae83WdmhabeaakiabcIcaOiabdwgaLnaaBaaaleaacqWGPbqAaeqaaOGaeiilaWIaemyzau2aaSbaaSqaaiabdQgaQbqabaGccqGGPaqkaSqaaiabdQgaQjabg2da9iabigdaXaqaaiabd6eaobqdcqGHris5aaWcbaGaemyAaKMaeyypa0JaeGymaedabaGaemOta4eaniabggHiLdGcdaWadaqaamaalaaabaGaeyOaIyRaemyzau2aaSbaaSqaaiabdMgaPbqabaaakeaacqGHciITcqWF7oaBaaGaeyOeI0YaaSaaaeaacqGHciITcqWGLbqzdaWgaaWcbaGaemOAaOgabeaaaOqaaiabgkGi2kab=T7aSbaaaiaawUfacaGLDbaaaaa@71EF@

The leave-one-out cross-validation error vector can be rewritten using the "entry-wise" Hadamard product represented by the symbol ∘ in the Equation 12.

*e *= [*y *- *Hy*] ο [*dg*(*I *- *H*)]^ο-1^

In the Equation 12, *dg(·) *is an operator applicable on squared matrices and it results in a vector which the elements are the diagonal of the matrix on its argument. The Equation 12 is formed by two vectors and each component of the first vector is "point-wise" divided by the correspondent component of the second vector, where [v]∘−1=[1vi]
 MathType@MTEF@5@5@+=feaafiart1ev1aaatCvAUfKttLearuWrP9MDH5MBPbIqV92AaeXatLxBI9gBaebbnrfifHhDYfgasaacH8akY=wiFfYdH8Gipec8Eeeu0xXdbba9frFj0=OqFfea0dXdd9vqai=hGuQ8kuc9pgc9s8qqaq=dirpe0xb9q8qiLsFr0=vr0=vr0dc8meaabaqaciaacaGaaeqabaqabeGadaaakeaadaWadaqaaiabdAha2bGaay5waiaaw2faamaaCaaaleqabaGaeSigI8MaeyOeI0IaeGymaedaaOGaeyypa0ZaamWaaeaadaWccaqaaiabigdaXaqaaiabdAha2naaBaaaleaacqWGPbqAaeqaaaaaaOGaay5waiaaw2faaaaa@3A61@ is the Hadamard inverse operation. In order to simplify the equations' notation, the Equation 6 is rewritten as

*H *= H^
 MathType@MTEF@5@5@+=feaafiart1ev1aaatCvAUfKttLearuWrP9MDH5MBPbIqV92AaeXatLxBI9gBaebbnrfifHhDYfgasaacH8akY=wiFfYdH8Gipec8Eeeu0xXdbba9frFj0=OqFfea0dXdd9vqai=hGuQ8kuc9pgc9s8qqaq=dirpe0xb9q8qiLsFr0=vr0=vr0dc8meaabaqaciaacaGaaeqabaqabeGadaaakeaacuWGibasgaqcaaaa@2DD5@*W*

where H^
 MathType@MTEF@5@5@+=feaafiart1ev1aaatCvAUfKttLearuWrP9MDH5MBPbIqV92AaeXatLxBI9gBaebbnrfifHhDYfgasaacH8akY=wiFfYdH8Gipec8Eeeu0xXdbba9frFj0=OqFfea0dXdd9vqai=hGuQ8kuc9pgc9s8qqaq=dirpe0xb9q8qiLsFr0=vr0=vr0dc8meaabaqaciaacaGaaeqabaqabeGadaaakeaacuWGibasgaqcaaaa@2DD5@ = [*W *+ *λD*^*T*^*D*]^-1^. Determining the derivative of the error with respect to *λ*, one thus obtains

∂e∂λ=([H^(∂H^−1∂λ)H^W]y)∘[dg(I−H)]∘−1−(dg[H^(∂H^−1∂λ)H^W])∘(y−Hy)∘[dg(I−H)]∘−2
 MathType@MTEF@5@5@+=feaafiart1ev1aaatCvAUfKttLearuWrP9MDH5MBPbIqV92AaeXatLxBI9gBaebbnrfifHhDYfgasaacH8akY=wiFfYdH8Gipec8Eeeu0xXdbba9frFj0=OqFfea0dXdd9vqai=hGuQ8kuc9pgc9s8qqaq=dirpe0xb9q8qiLsFr0=vr0=vr0dc8meaabaqaciaacaGaaeqabaqabeGadaaakeaadaWcaaqaaiabgkGi2kabdwgaLbqaaiabgkGi2IGaciab=T7aSbaacqGH9aqpdaqadaqaamaadmaabaGafmisaGKbaKaadaqadaqaamaalaaabaGaeyOaIyRafmisaGKbaKaadaahaaWcbeqaaiabgkHiTiabigdaXaaaaOqaaiabgkGi2kab=T7aSbaaaiaawIcacaGLPaaacuWGibasgaqcaiabdEfaxbGaay5waiaaw2faaiabdMha5bGaayjkaiaawMcaaiablIHiVnaadmaabaGaemizaqMaem4zaC2aaeWaaeaacqWGjbqscqGHsislcqWGibasaiaawIcacaGLPaaaaiaawUfacaGLDbaadaahaaWcbeqaaiablIHiVjabgkHiTiabigdaXaaakiabgkHiTmaabmaabaGaemizaqMaem4zaC2aamWaaeaacuWGibasgaqcamaabmaabaWaaSaaaeaacqGHciITcuWGibasgaqcamaaCaaaleqabaGaeyOeI0IaeGymaedaaaGcbaGaeyOaIyRae83UdWgaaaGaayjkaiaawMcaaiqbdIeaizaajaGaem4vaCfacaGLBbGaayzxaaaacaGLOaGaayzkaaGaeSigI82aaeWaaeaacqWG5bqEcqGHsislcqWGibascqWG5bqEaiaawIcacaGLPaaacqWIyiYBdaWadaqaaiabdsgaKjabdEgaNnaabmaabaGaemysaKKaeyOeI0IaemisaGeacaGLOaGaayzkaaaacaGLBbGaayzxaaWaaWbaaSqabeaacqWIyiYBcqGHsislcqaIYaGmaaaaaa@7CA5@

Equation 14 can be simplified and rewritten as:

∂e∂λ=[[H^(∂H^−1∂λ)H]y−(dg[H^(∂H^−1∂λ)H])∘e]∘[dg(I−H)]∘−1
 MathType@MTEF@5@5@+=feaafiart1ev1aaatCvAUfKttLearuWrP9MDH5MBPbIqV92AaeXatLxBI9gBaebbnrfifHhDYfgasaacH8akY=wiFfYdH8Gipec8Eeeu0xXdbba9frFj0=OqFfea0dXdd9vqai=hGuQ8kuc9pgc9s8qqaq=dirpe0xb9q8qiLsFr0=vr0=vr0dc8meaabaqaciaacaGaaeqabaqabeGadaaakeaadaWcaaqaaiabgkGi2kabdwgaLbqaaiabgkGi2IGaciab=T7aSbaacqGH9aqpdaWadaqaamaadmaabaGafmisaGKbaKaadaqadaqaamaalaaabaGaeyOaIyRafmisaGKbaKaadaahaaWcbeqaaiabgkHiTiabigdaXaaaaOqaaiabgkGi2kab=T7aSbaaaiaawIcacaGLPaaacqWGibasaiaawUfacaGLDbaacqWG5bqEcqGHsisldaqadaqaaiabdsgaKjabdEgaNnaadmaabaGafmisaGKbaKaadaqadaqaamaalaaabaGaeyOaIyRafmisaGKbaKaadaahaaWcbeqaaiabgkHiTiabigdaXaaaaOqaaiabgkGi2kab=T7aSbaaaiaawIcacaGLPaaacqWGibasaiaawUfacaGLDbaaaiaawIcacaGLPaaacqWIyiYBcqWGLbqzaiaawUfacaGLDbaacqWIyiYBdaWadaqaaiabdsgaKjabdEgaNnaabmaabaGaemysaKKaeyOeI0IaemisaGeacaGLOaGaayzkaaaacaGLBbGaayzxaaWaaWbaaSqabeaacqWIyiYBcqGHsislcqaIXaqmaaaaaa@6780@

As proposed in [9], in order to speed up the process, we optimize *λ *= 10^*ψ *^in the Equation 6, which results the derivative of the inverse of the hat matrix to

∂H^−1∂λ=dλdψ(DTD),
 MathType@MTEF@5@5@+=feaafiart1ev1aaatCvAUfKttLearuWrP9MDH5MBPbIqV92AaeXatLxBI9gBaebbnrfifHhDYfgasaacH8akY=wiFfYdH8Gipec8Eeeu0xXdbba9frFj0=OqFfea0dXdd9vqai=hGuQ8kuc9pgc9s8qqaq=dirpe0xb9q8qiLsFr0=vr0=vr0dc8meaabaqaciaacaGaaeqabaqabeGadaaakeaadaWcaaqaaiabgkGi2kqbdIeaizaajaWaaWbaaSqabeaacqGHsislcqaIXaqmaaaakeaacqGHciITiiGacqWF7oaBaaGaeyypa0ZaaSaaaeaacqWGKbazcqWF7oaBaeaacqWGKbazcqWFipqEaaGaeiikaGIaemiraq0aaWbaaSqabeaacqWGubavaaGccqWGebarcqGGPaqkcqGGSaalaaa@41CC@

where dλdψ=10ψln⁡10
 MathType@MTEF@5@5@+=feaafiart1ev1aaatCvAUfKttLearuWrP9MDH5MBPbIqV92AaeXatLxBI9gBaebbnrfifHhDYfgasaacH8akY=wiFfYdH8Gipec8Eeeu0xXdbba9frFj0=OqFfea0dXdd9vqai=hGuQ8kuc9pgc9s8qqaq=dirpe0xb9q8qiLsFr0=vr0=vr0dc8meaabaqaciaacaGaaeqabaqabeGadaaakeaadaWcaaqaaiabdsgaKHGaciab=T7aSbqaaiabdsgaKjab=H8a5baacqGH9aqpcqaIXaqmcqaIWaamdaahaaWcbeqaaiab=H8a5baakiGbcYgaSjabc6gaUjabigdaXiabicdaWaaa@3C6A@. Substituting Equation 16 into 15 and then 15 into 11 results in the gradient of *IP *of *cvE*, and allows recursive determination of *λ *in Equation 10. To prevent problems caused by an amplitude shift in the signal, we found it advantageous to normalize the signal to a linear scale with the range [0, 1].

### Signal segmentation

The Whittaker's smoother assumes an invariant noise structure throughout the signal [10]. This assumption is often not valid for biological time series such as those measured in metabolic profile analyses. To overcome this problem, the proposed smoothing procedure includes a process for segmenting the time series into windows with similar noise structure.

The procedure starts by smoothing the entire signal and calculating *cvEE*. This quantity is determined with a static kernel size *σ*_*s *_that is estimated from the raw data series and will later be used as a stop criterion. In the next step, the signal is divided into two windows. Window 1 (left) contains initially only the first four points, while window 2 (right) consists of the complementary signal. For each window, the smoother parameters are optimized using the method described in the previous section. Next, the cost function

cf=‖Hσww−Hσw0‖⤢,w=1,2
 MathType@MTEF@5@5@+=feaafiart1ev1aaatCvAUfKttLearuWrP9MDH5MBPbIqV92AaeXatLxBI9gBaebbnrfifHhDYfgasaacH8akY=wiFfYdH8Gipec8Eeeu0xXdbba9frFj0=OqFfea0dXdd9vqai=hGuQ8kuc9pgc9s8qqaq=dirpe0xb9q8qiLsFr0=vr0=vr0dc8meaabaqaciaacaGaaeqabaqabeGadaaakeaacqWGJbWycqWGMbGzcqGH9aqpdaqbdaqaaiabdIeainaaDaaaleaaiiGacqWFdpWCdaWgaaadbaGaem4DaChabeaaaSqaaiabdEha3baakiabgkHiTiabdIeainaaDaaaleaacqWFdpWCdaWgaaadbaGaem4DaChabeaaaSqaaiabicdaWaaaaOGaayzcSlaawQa7aiaaywW6cqGGSaalcqWG3bWDcqGH9aqpcqaIXaqmcqGGSaalcqaIYaGmaaa@47FE@

is evaluated, where ||·|| signifies the absolute value. Hσww
 MathType@MTEF@5@5@+=feaafiart1ev1aaatCvAUfKttLearuWrP9MDH5MBPbIqV92AaeXatLxBI9gBaebbnrfifHhDYfgasaacH8akY=wiFfYdH8Gipec8Eeeu0xXdbba9frFj0=OqFfea0dXdd9vqai=hGuQ8kuc9pgc9s8qqaq=dirpe0xb9q8qiLsFr0=vr0=vr0dc8meaabaqaciaacaGaaeqabaqabeGadaaakeaacqWGibasdaqhaaWcbaacciGae83Wdm3aaSbaaWqaaiabdEha3bqabaaaleaacqWG3bWDaaaaaa@32E2@ is the minimal *cvEE *found for window *w*, using kernel size *σ*_*w*_, and Hσw0
 MathType@MTEF@5@5@+=feaafiart1ev1aaatCvAUfKttLearuWrP9MDH5MBPbIqV92AaeXatLxBI9gBaebbnrfifHhDYfgasaacH8akY=wiFfYdH8Gipec8Eeeu0xXdbba9frFj0=OqFfea0dXdd9vqai=hGuQ8kuc9pgc9s8qqaq=dirpe0xb9q8qiLsFr0=vr0=vr0dc8meaabaqaciaacaGaaeqabaqabeGadaaakeaacqWGibasdaqhaaWcbaacciGae83Wdm3aaSbaaWqaaiabdEha3bqabaaaleaacqaIWaamaaaaaa@3259@ is the entropy of the null vector with the same kernel size *σ*_*w *_estimated from the points in the respective window. For each iteration, window 1 is increased by one time point and window 2 is correspondingly decreased by the same point, and the process of parameter optimization and evaluation of the cost function *cf *is performed again. After *N*-7 iterations the signal is completely scanned and the entropy information of the windows is evaluated (Figure 1). The window with minimal cost function *cf *and its complementary window are chosen, and the *cvEE *is updated for this new smoothed configuration. The signal is definitely broken into two windows if the new *cvEE *is lower than the current *cvEE*, which had been obtained before of the scanning process. The same search process for a break point (minimal *cf *point) repeats inside each of the new two windows. The recursion proceeds until the scanning of all windows produces *cvEE *values that are above the one obtained when the window was segmented. To remove the effect of the kernel size, the entropy values are considered in the cost function *cf *as the signal's information and referenced by the minimal possible information measured with the same space metric, the kernel size *σ*_*w*_. This normalization removes the bias towards small windows, which would result in extraction of the noise through over-fitting. In summary, after each scanning run, the algorithm creates two new windows for each current window if the *cvEE *of the entire signal is minimized, and otherwise terminates the recursive segmentation. The windows with minimal entropy match with the assumption of "constant measure of precision" made by Whittaker in the smoother equation [10]. In the unlikely event that more than one window reaches the same minimum *cvEE*, which would make the cost function zero, the break point will be selected as the one where the complementary window has the minimum *cvEE *(Figure 1c). As the scanning process goes progressively through the signal where the two windows have one fix point (the first point for the left window and the last point for the right window), the algorithm works well only with signal that presents a gradual changes on the noise structure, no matters in which direction. One general solution could be built by moving the fix points of the windows, scanning all the possible segments of the signal. It would require a great computational power, but fortunately this solution is out of our main purpose. Most biological time series have a noise behavior addressed here that makes the tool described sufficiently useful for application on metabolomic profiles. Tests with a different synthetics signal were performed [see Additional file 1]. The segmentation algorithm together with the kernel density estimation (in the parameters optimization procedure) comes with a significant computational cost. However, this cost allows independent model identification for each metabolic in the time series. The resulting parallel parameterization allowed by an efficient numerical decoupling translates into immense net computational gains even for S-systems models (or other systems of coupled differential equations) with as few as 3 variables.

## Availability and requirements

The library implementing the filter described in this report is provided both with open source (MathWorks Matlab) and as a stand alone application. The library is provided at  with free access and use, under a GNU GPL license. It can also be conveniently obtained as a module of the Bioinformatics Station resource .

## Authors' contributions

MV developed and implemented the algorithm. JSA devised and supervised the theoretical and numerical components of the study. ATRV and HS supervised the biological analysis. CCHB designed and supervised the numerical tests. SV advised in the information theory analysis. EBO provided critical insight in pathway analysis and interpretation of results. All authors contributed to preparation of the manuscript.

## Supplementary Material

Additional file 1 Tests and comparisons. Comparisons with Savitzky-Golay filter and tests with synthetic signals. Click here for file
