# Supplementary material for: Automated smoother for the numerical decoupling of dynamics models
Source: BMC Bioinformatics. 2007 Aug 21;8:305. doi: 10.1186/1471-2105-8-305 (PMC2041957; doi:10.1186/1471-2105-8-305)
Supplement: Additional file 1 — Tests and comparisons. Comparisons with Savitzky-Golay filter and tests with synthetic signals. [file 1471-2105-8-305-S1.doc]

Supplementary material to

*Automated smoother for the numerical decoupling of dynamics models*

Marco Vilela, Carlos C.H. Borges, Susana Vinga, Ana Tereza R. Vasconcelos, Helena Santos, Eberhard O. Voit and Jonas S. Almeida

## Tests and comparisons

In order to provide an assessment of the proposed method it was applied to a test set of synthetics time series. The very last test uses real data. This supplementary material comprises two sections. In the first one the performance of the proposed method is assessed by comparing with the popular Savitzky-Golay (SG) filter. To avoid an unfair scenario for SG filter, the test time series will have an invariant noise. On the contrary, in the second part of this supplementary material, the focus will be on segmentation (windowing) procedure to assess if shifts in noise structure are correctly identified by the proposed method.

1. Comparison with Savitzky-Golay filter

The Savitzky-Golay (SG) filter has been used as a smoothing tool for over 40 years. Basically, the SG filter performs a polynomial regression on a pre-determined window of the signal and replaces the original central point with the one obtained by regression. After that, the window shift by one point forward and the same process is repeated, and so on.

In order to compare the two smoothing methods, we applied both smoothers to a synthetic noise time series where the noise-free signal was known. The same optimization criterion was applied for both smoothers –the cross-validation error entropy. The data was corrupted with different levels of Gaussian noise. The levels of noise (variance) were kept constant throughout the signal. For each level we performed 50 “runs”. The Mean Squared Error (MSE) was then evaluated using the noise-free curve and its mean can be seen in Table 1.

| Noise variance | SG filter MSE | Whittaker Filter MSE |
| --- | --- | --- |
| 0.1 | 0.000886 | 0.000778 |
| 0.2 | 0.002991 | 0.00284 |
| 0.3 | 0.006149 | 0.005701 |
| 0.4 | 0.010315 | 0.009533 |

Table 1 – Comparison between the proposed extension of the Whittaker filter and Savitzky-Golay filter using the Cross-Validation Error Entropy as the optimization criteria.

In an unfair comparison, the SG filter was parameterized using the MSE between the known original data points and the filter output, which makes it an optimal parameterization. The Whittaker filter with the proposed optimization process (Minimal Cross-Validation Error Entropy - MCVEE) was applied in the same time series. The result is shown in Figure 1.

The Figure 1b shows a “zoom in” on the result of the two filters. It is clearly apparent in that figure that the SG filter result extracts a signal with higher roughness in comparison to the Whittaker filter, even when the MSE of the SG filter is smaller (see Figure 1 legend). This result is amplified in its temporal derivative, as shown in the Figure 2.


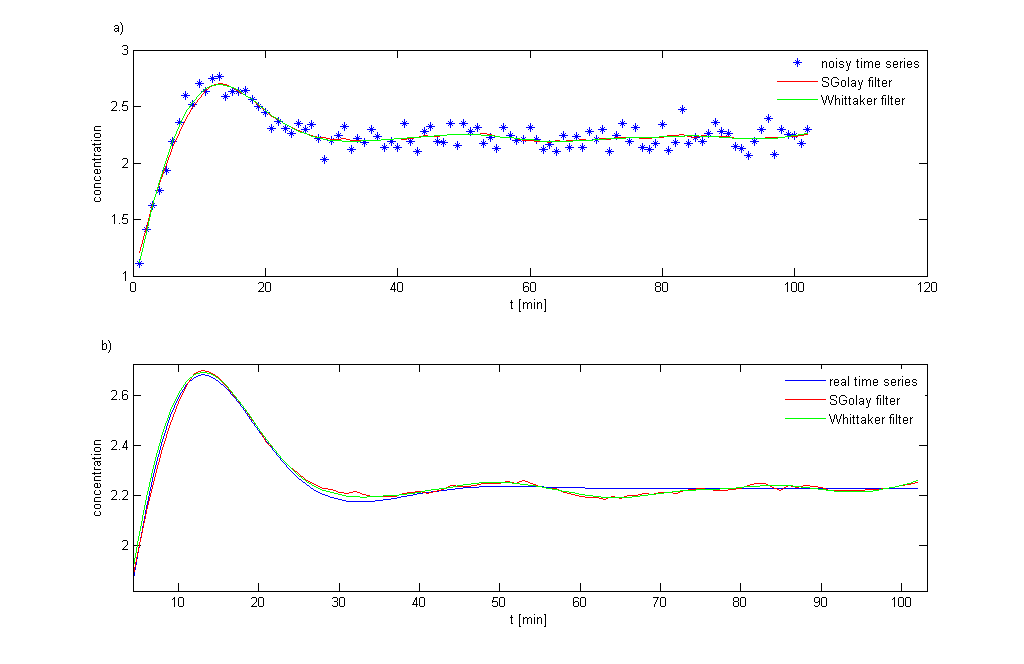


Figure 1 – Smoothers comparison. a) results of the Whittaker and SG filters applied on synthetic time series with Gaussian noise with variance 0.1. b) “Zoom in” on the smoothers results. MSE obtained with the real time series (blue) – Wf=0.000378; SGf=0.000347.


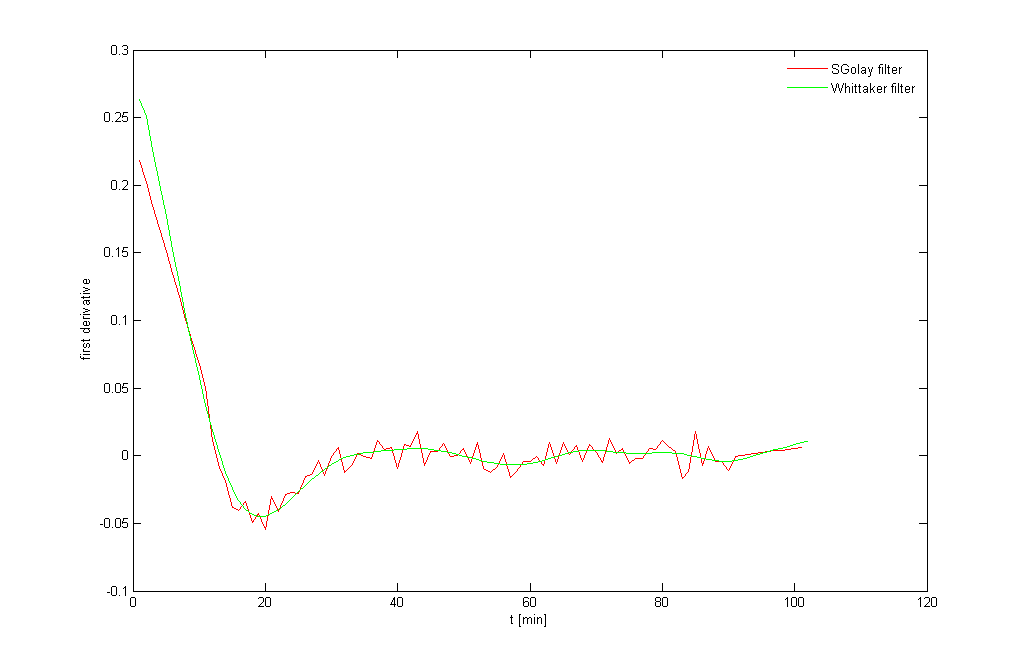


Figure 2 – Temporal derivative of the Savitzky-Golay (red) and Whittaker (green) smoothers. The roughness of the result of the SG filter is amplified in its derivative.

2. Segmentation process

Several time series with different levels of noise throughout the signal were built to test the segmentation algorithm. These tests show that the proposed method is effective in accommodating varying noise structures therefore avoiding biasing local signal extraction. Variation in noise levels throughout biomolecular time series is a common observation. In the Discussion section it was hypothesized that this reflects variation of the molecular machinery producing the signal at different stages of the process tracked by the series. It is also suggested here another common occurrence – shifts in the noise level caused by changing performance of the monitoring equipment may be another situation where this procedure may prove invaluable. The results presented in the Figures 3, 4, 5 show how shift in noise levels, independently of the shape of the signal, are correctly identified.


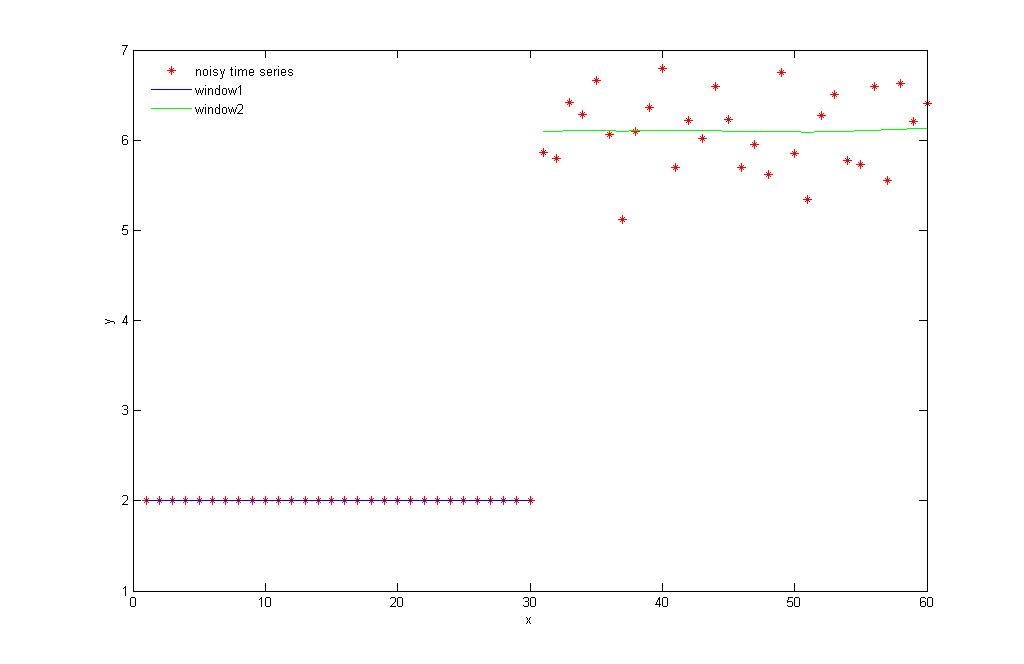


Figure 3 – Test curve 1. Two constant functions (step function) with Gaussian noise of variance 0.1 on the higher level. This is a very basic test because of the clear discontinuity of the signal at the same point in time where the noise structure shifts.


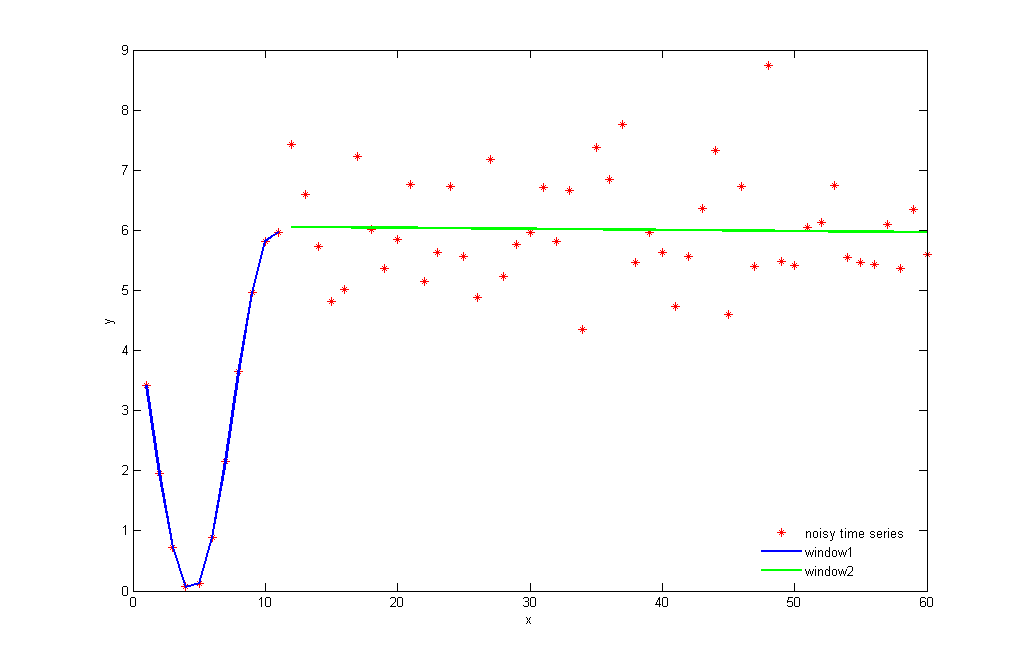


Figure 4 – Test curve 2. A polynomial (blue) + constant (green) with Gaussian noise of variance 0.2 on the constant segment. This is a harder test than the previous example because the noise shits at x=10 but the signal shows no interruption.


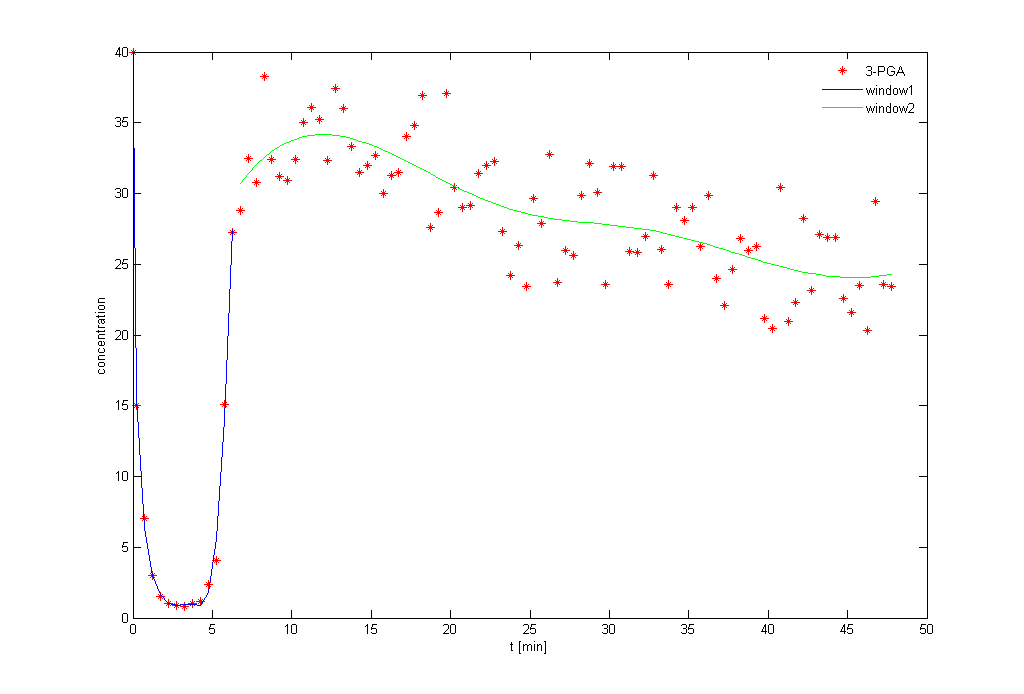


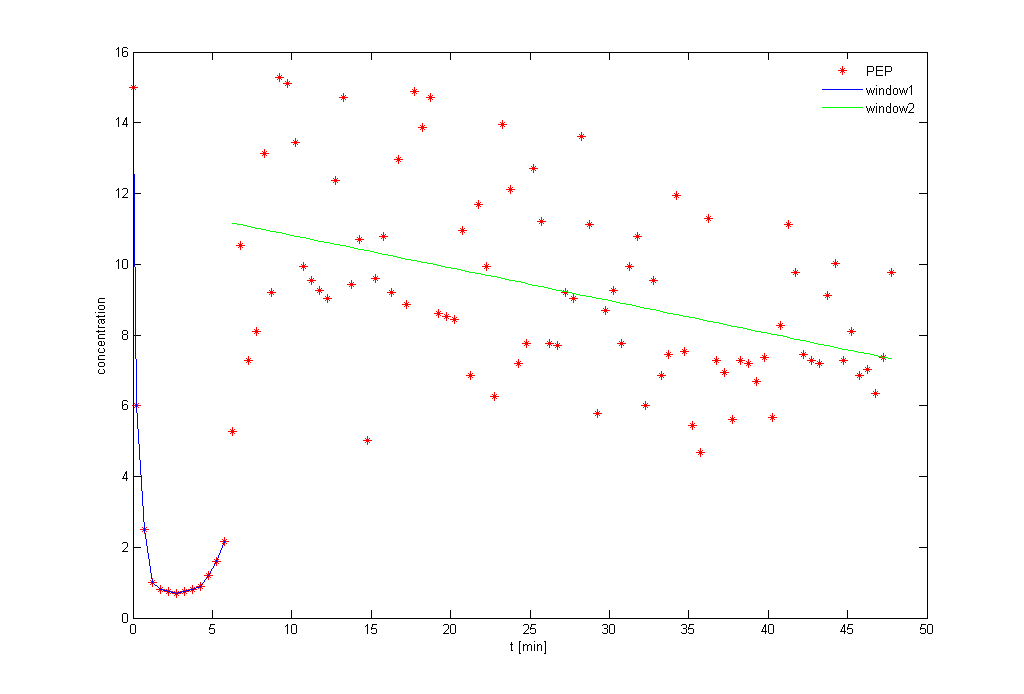


Figure 5 – Test curve 3 and 4. The last test uses real data, produced by *invivo* NMR as described in the main text of the report. Here we have an example of a continuous with a shift noise level between t~6.5 min. The noise level in the second portion of the series is much higher and much more skewed in the second example. As it is apparent in length and shape of the signal extracted (green), the noise shift is correctly identified both times and higher level of noise handled by extracting a featureless linear signal.
